# Bimetallic CuNi Nanoparticle Formation: Solution Combustion Synthesis and Molecular Dynamic Approaches

**DOI:** 10.1021/acs.inorgchem.4c04260

**Published:** 2024-12-16

**Authors:** Valentin Romanovski, Nickolay Sdobnyakov, Sergey Roslyakov, Andrei Kolosov, Kirill Podbolotov, Kseniya Savina, Witold Kwapinski, Dmitry Moskovskikh, Alexander Khort

**Affiliations:** † Department of Materials Science and Engineering, University of Virginia, Charlottesville, Virginia 22908, United States; ‡ Science and Research Centre of Functional Nano-Ceramics, National University of Science and Technology “MISIS”, Moscow 119049, Russia; § Department of General Physics, Tver State University, Tver 170002, Russia; ∥ Physical-Technical Institute of the National Academy of Sciences of Belarus, Minsk 220141, Belarus; ⊥ Department of Chemical Sciences, Bernal Institute, University of Limerick, Limerick V94 T9PX, Ireland; # Division Surface and Corrosion Science, KTH Royal Institute of Technology, 114 28 Stockholm, Sweden

## Abstract

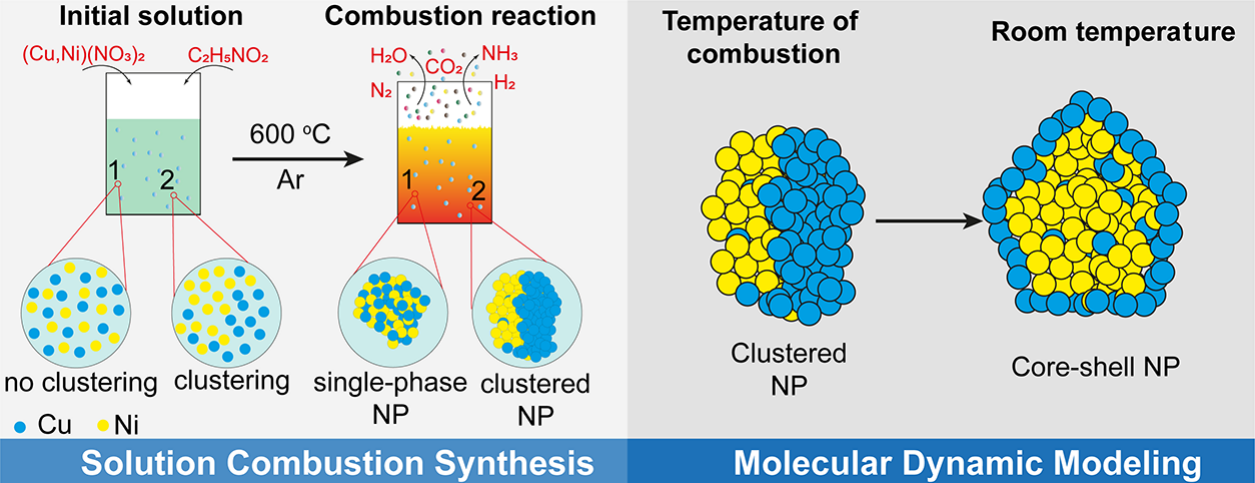

Nanomaterials are vital in catalysis, sensing, energy storage, and biomedicine and now incorporate multiprincipal element materials to meet evolving technological demands. However, achieving a uniform distribution of multiple elements in these nanomaterials poses significant challenges. In this study, various Cu–Ni compositions were used as a model system to investigate the formation of bimetallic nanoparticles by employing computer simulation molecular dynamics methods and comparing the results with observations from solution-combustion-synthesized materials of the same compositions. The findings reveal the successful synthesis of 12–18 nm bimetallic Cu–Ni nanoparticles with high phase homogeneity, alongside phase-segregated nanoparticles predicted by molecular dynamics simulations. Based on the comparison of the experimental and computational data, a possible scenario for phase segregation during the synthesis was proposed. It includes clustering of the atoms of the same type in an initial solution or the stage of gel formation and further developing segregation during the combustion/cooling stage. The research concludes that early synthesis stages, including particle preformation, significantly influence the phase homogeneity of multiprincipal element alloys. This study contributes to understanding nanomaterial formation, offering insights for improved alloy synthesis and enhanced functionalities in advanced applications.

## Abstract 2

In our research, we investigated the formation of the bimetallic CuNi nanoparticles of different compositions both using experimental solution combustion synthesis (SCS) and molecular dynamic modeling approaches. We found that using SCS allows obtaining high-homogeneous CuNi bimetallic nanoparticles, alongside phase-segregated nanoparticles predicted by molecular dynamics simulations. Based on the comparison of the experimental and computational data, we proposed a possible scenario for phase segregation during the synthesis.

## 1 Introduction

In recent decades, nanomaterials have proven to be exceptionally valuable across a wide range of advanced applications. The key driver behind their enhanced functional properties lies in their unique morphology, where a significant portion of atoms are on or near the surface regions of the nanoparticle (NP), creating structures with high free energy and defining distinct nanospecific properties. Notably, metallic and oxide NPs exhibit superior catalytic activity in various reactions,^1−3^ used as sensing materials in advanced semiconductor sensors,^4−6^ electrodes for supercapacitors and batteries,^7,8^ biomedical applications,^9,10^ and more.^11−13^ However, the rigorous pace of technological advancement demands materials with even greater functional characteristics to align with the ongoing trends for device and instrument miniaturization. A cutting-edge approach to achieving this is through alloying and doping. In this context, the development of multiprincipal element materials (MPEMs) has emerged as highly successful.^14,15^

MPEMs typically contain multiple elements that may not be in equal concentrations but distributed with equal probability within the same crystalline sublattice according to their respective concentrations.^16,17^ Such a combination of elements gives rise to entropy-induced effects,^18,19^ resulting in materials that for instance display enhanced catalytic activity,^20−22^ energy storage,^23,24^ and superior phase stability in corrosive environments^25−27^ compared to their monoelement counterparts. Nevertheless, synthesizing MPEM nanomaterials poses challenges, primarily due to the difficulty in controlling the mixing of elements and ensuring their uniform distribution within the structure.^28−30^ For example, it is well-known that Cu tends to segregate from multicomponent nanoalloys, forming separate crystal phases.^17,26^ Therefore, it is crucial to gain a comprehensive understanding of the processes involved in the formation of nanoparticles with multiple principal elements. This knowledge is essential for overcoming the complexities associated with element mixing and achieving a homogeneous distribution, ultimately unlocking the full potential of MPEMs in advanced applications.

Among the intricate processes shaping the properties and applications of nanoparticles with multiple principal elements, coalescence emerges as a pivotal phenomenon, offering insights into the mechanisms of growth, aggregation, and phase transformation. Coalescence, the process by which nanoparticles merge to form larger structures through the reduction of surface energy, is a critical phenomenon in nanomaterials science. This process is central to understanding the behavior of nanoparticles during growth and aggregation and has significant implications for various applications, including catalysis, energy storage, and nanostructure fabrication. This process is essential in determining the size, shape, and properties of nanoparticles, impacting applications from catalysis to materials engineering. Coalescence has gained attention as a method for creating nanomaterials with tailored properties, highlighting its industrial relevance. Studies show that cluster sources influence the growth and composition of bimetallic nanoparticles, underscoring the need for precise synthesis control.^31^ The initial structure and orientation of nanoparticles also affect their aggregation and coalescence pathways.^32,33^ Experimental and theoretical studies highlight that nanoparticle coalescence is significantly influenced by crystallographic orientation, with findings indicating a two-step process involving the reorientation of neighboring particles followed by complete or partial merging, as dictated by lattice alignment.^34^ These factors highlight the complexity of the nanoparticle interactions.

Theoretical and computational approaches have advanced our understanding of coalescence. Simulations offer insights into nanoparticle growth, coalescence, and phase transformations under varying conditions.^35,36^ Techniques like density functional tight-binding have revealed the outcomes of nanoparticle collisions, providing predictive insights into postcollision interactions.^37^ Recent advancements, such as well-tempered metadynamics, explore the free energy landscapes of coalescing nanoparticles, capturing rare events crucial to understanding nanoparticle behavior.^38,39^ Metadynamics has also clarified nanoparticle transformations and core–shell formation in bimetallic nanoparticles.^40,41^ Comparative studies of computational techniques, particularly for analyzing thermal properties in systems like Co/Au nanoalloys, have further enriched understanding.^42^ Thermal effects are critical in coalescence, influencing the structural evolution and the final properties of nanostructures. For instance, cooling rates and thermal dynamics determine the outcomes of coalescence in bimetallic nanoclusters.^43−45^ Innovative techniques, such as room-temperature assembly of Cu–Ag nanoparticles, demonstrate the practical applications of coalescence in nanoscale manufacturing.^46^ These studies emphasize the theoretical and practical importance of nanoparticle coalescence, as both a natural process and a synthesis method, driving advancements in nanomaterial science and industrial applications.

In this study, we used bimetallic Cu–Ni NPs with varying Cu-to-Ni ratios as model systems because these systems are quite commonly used in various industrial applications, they are relatively simple, and Cu and Ni form continuous series of solid solutions. Our objective was to explore the processes involved in the formation of bimetallic NPs through computer simulations under the likely conditions of NP synthesis. These simulation results were then compared with experimental observations for NPs of identical compositions obtained via solution combustion synthesis (SCS).

The chosen synthesis method is characterized by the rapid and highly energetic redox reaction of initial components mixed in a solution. This approach ensures a highly homogeneous mixing of elements prior to the start of the reaction. This is achieved by mixing soluble chemicals in water-based solutions, which forms metastable Me-based complexes with organic fuel/reducer as a result of drying and xerogel formation.^47,48^ The combination of fast heating during the self-propagating redox exothermic reaction between oxidizer (metal/nitrates) and reducer (organic fuel) and subsequent fast cooling in postprocessing prevents the nanoparticles from phase segregation. Notably, this method has proven successful in synthesizing both mono- and multimetallic NPs before.^49−53^ In this scenario, achieving the reduction of salts to their metallic state hinges on selecting the appropriate combination of salts and fuel. Through the decomposition of these components, a reducing atmosphere formed in situ, promoting the transformation.

Current trends underscore the adoption of high-precision computational methods to predict the bimetallic NP structure, properties, and synthesis processes. Various simulation methods, including quantum theoretical calculations, Monte Carlo (MC), and molecular dynamics (MD) methods, play a crucial role in comprehending bimetallic NP synthesis processes.^53−58^ Each method possesses distinct advantages and limitations. Quantum mechanics is introduced indirectly through interaction potentials. However, realistic results often require adjusting these potentials based on experimental data. The MC method and its variants are widely employed in computational studies, particularly in the investigation of isolated nanoparticles and nanostructured materials. However, it has the limitation of not providing insights into the temporal evolution of a physical process. In contrast, the MD method offers an advantage by enabling the acquisition of information about not only equilibrium configurations but also intermediate states. MD, grounded in classical physics, encounters challenges in describing quantum effects and necessitates specific knowledge of particle interactions, employing different models for diverse cases. However, MD methods excel in handling large nanosystems with 10^5^–10^7^ atoms, enabling the solution of Newtonian equations of particle motion. This permits the calculation of a system’s evolution over a set number of time steps, providing information at each step about particle position, speed, kinetic and potential energy, and more. All thermodynamic characteristics of the system can be determined without using any additional parameters. The accuracy of MD simulations hinges on the reliability of the interatomic potential models employed. In this study, the many-body-embedded-atom method (EAM) potential was used. EAM potentials have demonstrated success in describing various properties of metallic systems, including lattice constants, vacancy formation energies, and cohesive energies.^59^ Hence, for the computer simulations in this study, the MD method was selected.

Although the time scales of the NP formation, stabilization, and cooling for the MD simulation and SCS are considerably different, the comparison of the results of both the MD simulation and SCS nevertheless is practically interesting and gives a better understanding of the formation of NPs in Cu–Ni and other bimetallic and more complex MPEM systems.

The study’s findings not only deepen the general understanding of the formation of nanomaterials containing multiple principal elements but also contribute to a broader comprehension of the distinctive behavior and properties exhibited by MPEMs, in particular taking into account the influence of size effects and possible variations in composition and types of element distribution by volume (core–shell structure, onion-like structure, Janus structure, or uniform distribution).

## 2 Materials and Methods

### 2.1 Thermodynamic Calculations

Thermodynamic calculations (TCs) of exothermic processes involved in the synthesis were conducted using universal software ASTRA-4 (developed by Bauman Moscow State Technical University-MSTU). This software employs Gibbs energy minimization techniques and utilizes a comprehensive database containing parameters for inorganic materials to determine the final composition of the reaction. Input data included the initial chemical composition of the system and the specified thermodynamic regimes of reactions (in our case adiabatic, i.e., isobaric–isoenthalpic). As a result, we obtained the adiabatic temperature (*T*_ad_) and the equilibrium composition of the multicomponent multiphase system at *T*_ad_.

### 2.2 Materials and Reagents

All chemicals were analytical-grade reagents (>99% purity, LLC “KHIMMED”) and used without any further purification.

Nickel nitrate hexahydrate (Ni(NO_3_)_2_·6H_2_O) and copper nitrate trihydrate (Cu(NO_3_)_2_·3H_2_O) were used as a metals atom source and oxidizer, while glycine (C_2_H_5_NO_2_, G) was used as fuels/reducer.

### 2.3 Synthesis Procedure

Cu–Ni NP samples with Cu-to-Ni molar ratios of 2:1, 1:1, and 1:2 (samples Cu2Ni, CuNi, and CuNi2, respectively) were synthesized by a modified SCS method using a mixture of metal nitrates with glycine. In all experiments, the fuel-to-oxidizer molar ratio φ was kept at 1.75, which was chosen based on our previous studies.^60^ The fuel-to-oxidizer ratio φ is defined such that φ = 1 corresponds to stoichiometric oxygen concentration, meaning that the initial mixture does not require atmospheric oxygen for the complete oxidation of the fuel, while φ > 1 (φ < 1) implies fuel-rich (or lean) conditions.

All samples were prepared according to the calculated compositions (Table S1) by the same procedure as follows. At first, the required amount of metal nitrates was dissolved in a minimum volume of hot distilled water. After that, the fuel was gradually added to the solution under constant stirring to get the required φ according to SCS reactions in eq 1:
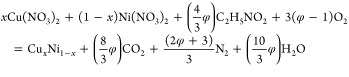
1

The obtained solution was rapidly dried in an oven at 373–393 K, until gel and foam had formed. The foam was ignited and burned in a preheated tube furnace at 873 K under an Ar atmosphere, leading to the formation of a fluffy powder, which was rapidly cooled to prevent metal oxidation.

### 2.4 Materials Characterization

The phase composition and structure of the obtained combustion products were characterized by X-ray diffraction (XRD) analysis using a Thermo ARL X’TRA (ThermoScientific) powder diffractometer with Cu Kα radiation (λ = 1.5405 Å), step size of 0.1°, and dwell time of 3 s. Rietveld refinements were conducted for the XRD profile analysis using HighScore Plus software. Pseudo-Voigt function was used for the peak profile refinement.

The microstructure and elemental composition of the powders were studied by using a JEOL JSM7600F scanning electron microscope (JEOL Ltd., Japan) equipped with an X-MAX 80 mm^2^ attachment (Oxford Instruments, U.K.) for X-ray microanalysis. SEM imaging and EDS analyses were performed at an acceleration voltage of 15 kV.

The fine structure was studied using a JEM-2100 Plus transmission electron microscope (JEOL Ltd., Japan) at an accelerating voltage of 200 kV. Image processing and grain size calculation were performed in ImageJ software.

Scanning/transmission electron microscopy (S/TEM) was carried out in an Osiris scanning transmission electron microscope (ThermoFisher Scientific) at an accelerating voltage of 200 kV. The device is equipped with a high-angle annular dark field (HAADF) detector and a Bruker energy-dispersive X-ray microanalysis (EDX) system (Bruker). Image acquisition and processing were performed using Digital Micrograph and TIA software. The samples were deposited on carbon-coated copper electron microscopy grids in their initial form without preliminary grinding.

### 2.5 Molecular Dynamic Simulation

The computer simulation of the formation of the Cu–Ni nanoparticles during the high-temperature synthesis was conducted by the MD method using the LAMMPS program^61^ with the parametrization of the embedded-atom potential.^62^ The applicability of the embedded-atom method (EAM) to binary nanoparticles has been evaluated, as demonstrated in previous studies.^63,64^ The temperature of the system was controlled using the classical Nosé–Hoover thermostat algorithm, implemented in the LAMMPS software package.^65^

Three sets of nanoparticles of 4.5–5.5 nm were used as simulation objects with a total number of atoms *N* = 6000 with the compositions Cu_3000_–Ni_3000_ (1:1), Cu_4000_–Ni_2000_ (2:1), and Cu_2000_–Ni_4000_ (1:2). In the initial configuration (Figure S1), the system consisted of two monometallic nanoparticles in contact with each other. According to the simulation conditions, the nanosystems initially relaxed during 15 ps at a temperature of *T*_s_ = 1700 K and then were cooled to a final temperature of *T*_f_ = 300 K at a rate of 1 K/ps. The value of the relaxation time is typical for considering the coalescence process^66^ that using synthesis temperature is almost doubled. This chosen cooling rate is instrumental in ensuring the thermal stability of the nanoparticles during the simulation. While experimental techniques cannot yet achieve the extremely high cooling rates used in molecular dynamics (MD) simulations, such as those exceeding 100 K/ps in some studies,^67,68^ these rates are essential for capturing rapid structural phenomena. In our simulation, a cooling rate of 1 K/ps was chosen as it represents a standard approach in MD, balancing the adequacy of results with computational efficiency while reflecting processes as closely as possible to experimental conditions. The starting temperature of 1700 K was chosen because it is close to the adiabatic temperature (*T*_ad_) of the system, which was obtained through thermodynamic calculations (see Table 1). This temperature is appropriate for observing the processes of interest, while maintaining computational feasibility. The selected size range for the nanoparticles is optimal for conducting computational experiments, balancing both accuracy and computational efficiency. Moreover, this size allows us to effectively study the segregation processes, which are the main focus of this work.

**Table 1:** Results of Thermodynamic Calculations of the Experimental Systems

			gas partial pressure, atm					solid products, %	
samples	adiabatic temperature *T*_ad_, K	products enthalpy, kJ/kg	CO	CO_2_	N_2_	H_2_O	gaseous products amount, mol/kg	Cu	Ni
Cu2Ni	1737	–4047.16	0.193	0.166	0.192	0.448	30.77	67	33
CuNi	1810	–4048.88	0.158	0.199	0.197	0.446	30.06	50	50
CuNi2	1866	–4079.27	0.121	0.233	0.201	0.443	29.37	33	67

It is also important to note that these nanoparticle sizes were selected because it is impossible to accurately determine the structure of individual agglomerates smaller than 5 nm. Therefore, in this case, the MD simulation plays a predictive role at these scales. In fact, the goal of the computer modeling in this work was to identify specific structural transformations corresponding to the smallest sizes observed in the experiment.

## 3 Results

### 3.1 Thermodynamic Calculations

The results of thermodynamic calculations of SCS for all samples (Table 1) show the calculated adiabatic combustion temperatures reached 1737–1866 K. However, in practice, the measured actual combustion temperature is significantly lower due to large heat losses related to the formation of a large amount of hot gases and heat transfer to the environment. For the CuNi-based system, for instance, the experimental global combustion temperature is ∼800 K.^60^ It should be noticed that calculated adiabatic temperatures (*T*_ad_) of the combustion reaction increase for samples with the increase of Ni content. This could be explained by the decrease of gaseous product amount along with the initial composition change. Considering the fact that the stoichiometric combustion reactions of nickel and copper nitrates are identical to each other, this may indicate differences in specific mechanisms of Ni(NO_3_)_2_–fuel and Cu(NO_3_)_2_–fuel interaction, i.e., the chemical reactivity of Cu and Ni.

Usually, for solid-state exothermic processes, enthalpy of reaction is the key factor influencing reaction temperature. However, in the case of heterogeneous SCS processes, the amount and type of gaseous products, as well as reaction kinetics, are equally important factors.

### 3.2 Materials Characterization

**Figure 1:**
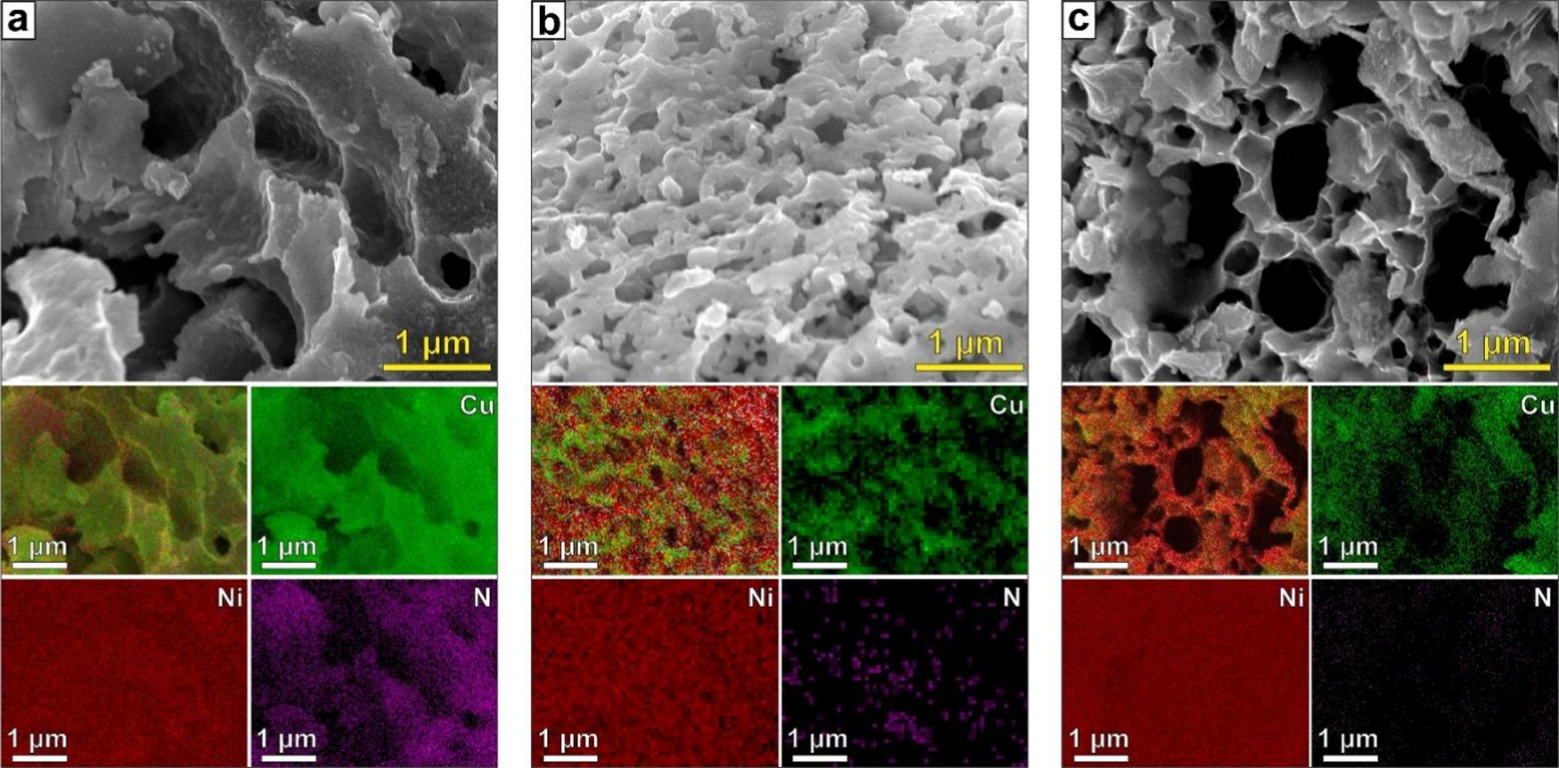
Morphology and chemical composition of Cu–Ni NPs. SEM images (top row) and elemental mapping (bottom row) of (a) Cu2Ni, (b) CuNi, and (c) CuNi2 NPs, showing the homogeneous distribution of Cu and Ni atoms.

The microstructure and chemical composition of the synthesized nanomaterials were investigated using SEM and EDX element mapping (Figure 1).

The analysis of the SEM images has revealed that the microstructure of Cu–Ni powders comprises highly porous agglomerates, a characteristic feature of materials obtained through the SCS.^47,48^ The agglomeration is a result of a self-propagated combustion reaction, leading to the formation of new layers of solid combustion products atop those already formed. Concurrently, the release of large amounts of hot gases leads to the formation of highly porous structures. These gases play a dual role. First, they facilitate efficient heat exchange between the constituents within the reactive system, fostering the propagation of a combustion wave. Second, they facilitate heat exchange between the reactive system and its surroundings. This dual functionality enhances the homogeneity of the final combustion product and crucially hinders the redistribution of elements within the crystal structure, thus mitigating the formation of specific elements clusters. The latter is especially important for the synthesis of monophase nanoalloys. Furthermore, this gas-mediated cooling mechanism serves to curtail the growth of the crystallites.

The EDX element mapping shows a highly homogeneous distribution of both Cu and Ni atoms, with the EDX-calculated Cu/Ni atom ratios 0.666/0.334, 0.503/0.497, and 0.331/0.669, for the Cu2Ni, CuNi, and CuNi2 samples, respectively. Moreover, the spectra of nitrogen atoms were also detected (Figure 1a–c). Nitrogen may originate from both the undecomposed initial chemicals and metal nitrides formed during the combustion reaction.^52,69^

**Figure 2:**
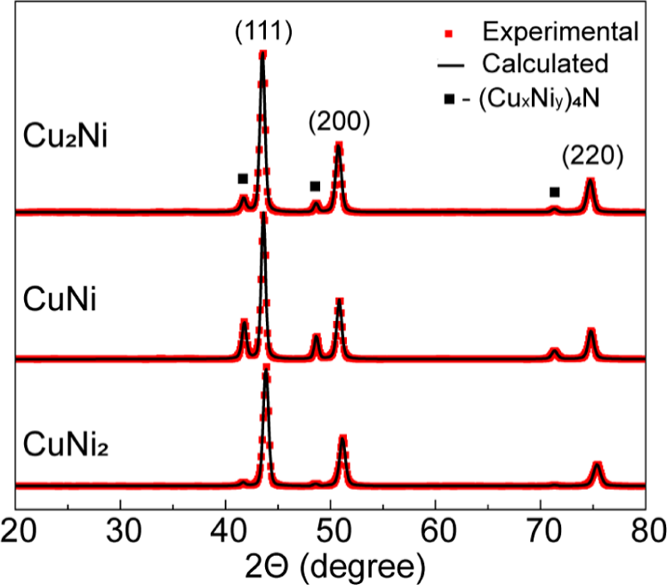
XRD patterns of SCS products.

The investigation of the phase composition of the synthesized materials was conducted using XRD. The typical XRD profiles of the materials and results of their analysis are presented in Figure 2 and Table 2. From the presented data, one can see that the main crystal phase in all samples is a bimetallic Cu–Ni alloy, with a distorted cubic structure (space group *Fm*3̅*m*) of nickel and copper and characteristic peaks at around 43.6–43.9° (111), 50.8–51.2° (002), and 74.7–75.4° (022). The value of the crystal cell parameter *a* in all cases is in-between values for metallic well-crystalline Cu (3.61 Å)^65^ and Ni (3.52 Å),^50^ with the largest crystal cell of the Cu2Ni NPs, and lowest the CuNi2 (Table 2). This confirms the formation of a single bimetallic crystal structure that contains both Cu and Ni atoms. Moreover, the peaks assigned to the (Cu_*x*_Ni_*y*_)_4_N phase were also detected in all cases. According to the results of XRD profiles analysis using the Rietveld refinement method, the content of the nitride phase is the highest in CuNi samples (18.3 wt %), as for Cu2Ni and CuNi2, the calculated amount of (Cu_*x*_Ni_*y*_)_4_N is 8.1 and 2.5 wt %, respectively (Table 2). No other crystalline phases were found.

**Table 2:** Crystal Lattice Parameters and Calculated Average Grain Size of the Samples

	phase composition		crystal lattice parameters			
samples	compound	wt %	space group	*a*, Å	*V*, Å^3^	*d*, nm
Cu2Ni	Cu_0.666_Ni_0.334_	91.9	*Fm*3̅*m* (cubic)	3.586	46.113	12.2
	(Cu_0.666_Ni_0.334_)_4_N	8.1	*Pm*-3m (cubic)	3.733	52.028	12.3
CuNi	Cu_0.331_Ni_0.669_	81.7	*Fm*3̅*m* (cubic)	3.585	46.062	17.6
	(Cu_0.331_Ni_0.669_)_4_N	18.3	*Pm*-3m (cubic)	3.735	52.110	17.5
CuNi2	Cu_0.331_Ni_0.669_	97.5	*Fm*3̅*m* (cubic)	3.557	45.015	17.1
	(Cu_0.331_Ni_0.669_)_4_N	2.5	*Pm*-3m (cubic)	3.728	51.812	14.4

As mentioned before, the Me_4_N crystal phase tends to form as an intermediate product during the SCS of the metallic nanomaterials.^52^ The nitride is thermally unstable and decomposes above 1023 K with the formation of a corresponding metallic phase. Thus, we can assume that the metal atom content and ratio are similar for both bimetallic alloy and nitride phases. Considering that there were no other phases but Cu_*x*_Ni_*y*_ and (Cu_*x*_Ni_*y*_)_4_N detected by XRD analysis and the results of the EDX elements content analysis, the chemical compositions of the final products of the SCS are Cu_0.666_Ni_0.334_ and (Cu_0.666_Ni_0.334_)_4_N, Cu_0.503_Ni_0.497_ and (Cu_0.503_Ni_0.497_)_4_N, and Cu_0.331_Ni_0.669_ and (Cu_0.331_Ni_0.669_)_4_N for Cu2N, CuNi, and CuNi2 samples, respectively.

The calculated sizes of the crystallites of the samples confirmed the formation of nanostructured materials. It can be seen that materials show a narrow mean crystallite size distribution across the samples from 12.2 nm for the Cu_0.666_Ni_0.334_, to 17.1–17.6 nm for the Cu_0.331_Ni_0.669_ and the Cu_0.503_Ni_0.497_ powders. It should also be noted that the crystallite sizes of Me nitride phases are very close to the sizes of corresponding alloys in cases of Cu2N and CuNi powders, which also confirmed the intermediate nature of the nitrides in the synthesis process. In the case of CuNi2 powders, however, the crystallite size of Me_4_N is ∼3 nm smaller compared to the alloy. This could be explained by the higher combustion temperature in the system, which corroborates the results of thermodynamic calculations (Table 1). We believe that in this case most of the nitride phase decomposed and only remnants of initially the largest nitride grains remained.

**Figure 3:**
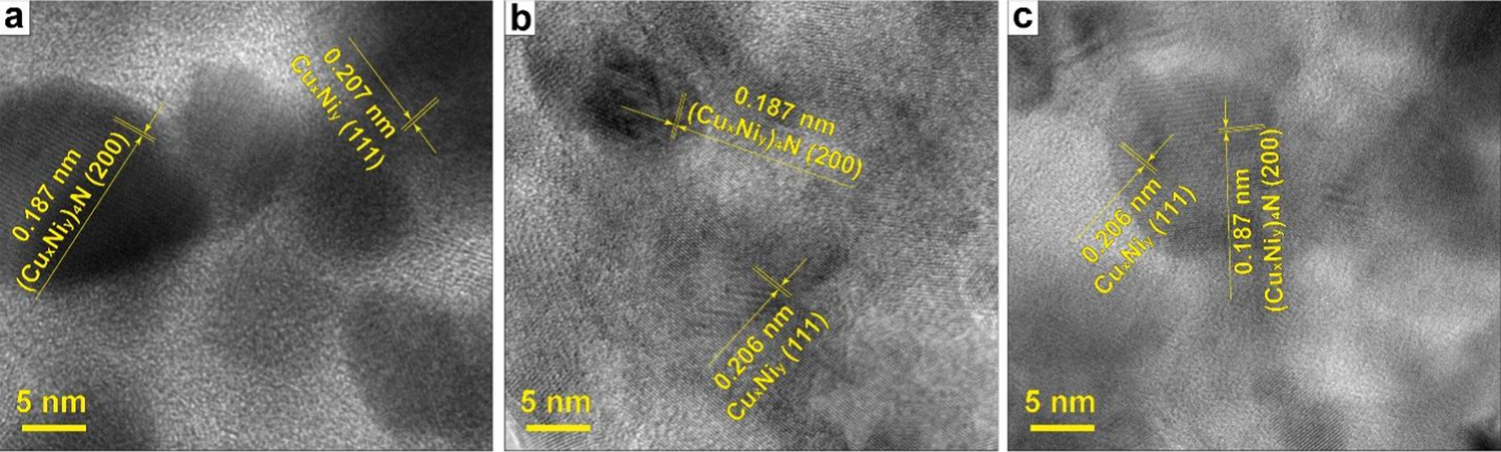
TEM images of Cu–Ni NPs. Images of (a) Cu2Ni, (b) CuNi, and (c) CuNi2 samples showing the presence of both bimetallic Cu–Ni and (Cu_*x*_Ni_*y*_)_4_N crystal phases.

A more detailed investigation of the features of the nanostructure and phase composition of the obtained nanomaterials was performed using the TEM technique. The typical TEM images of the grains of synthesized materials are presented in Figure 3.

From the images, one can see that in all the cases separate and well-defined grains of 10–20 nm are present in the sample, corroborating with XRD-calculated values. The grains are covered with thin graphene-like carbon films, typical for SCS-obtained metallic Cu- and Ni-contained nanomaterial, which was described in detail elsewhere,^70,71^ and thus is not discussed further in this paper. The analysis of the *d*-spacing of the separate grains revealed the presence of both metallic and nitride Cu–Ni crystal phases in all three samples (Figure 3a–c). The measured *d*-spacing value of the metallic Cu_*x*_Ni_*y*_ phase is 0.206–0.207 nm, again corroborates the XRD data and indicates the formation of the bimetallic crystals. In a few rare instances, we were able to observe NPs with segregated Cu–Ni crystal phases (Figures S2–S4).

Further investigation of the possible system behavior during the cooling process was performed by using MD simulations.

### 3.3 Results of Atomistic Simulations of Cu–Ni NP Formation

The MD method was used to simulate the behavior of the system containing clustered Cu and Ni atoms. The starting temperature *T*_s_ of 1700 K was selected as it is close to *T*_ad_ of the systems, obtained from thermodynamic calculations (Table 1). The final temperature of the simulation *T*_f_ is 300 K, close to the room temperature, where the system is considered mostly stabilized.

**Figure 4:**
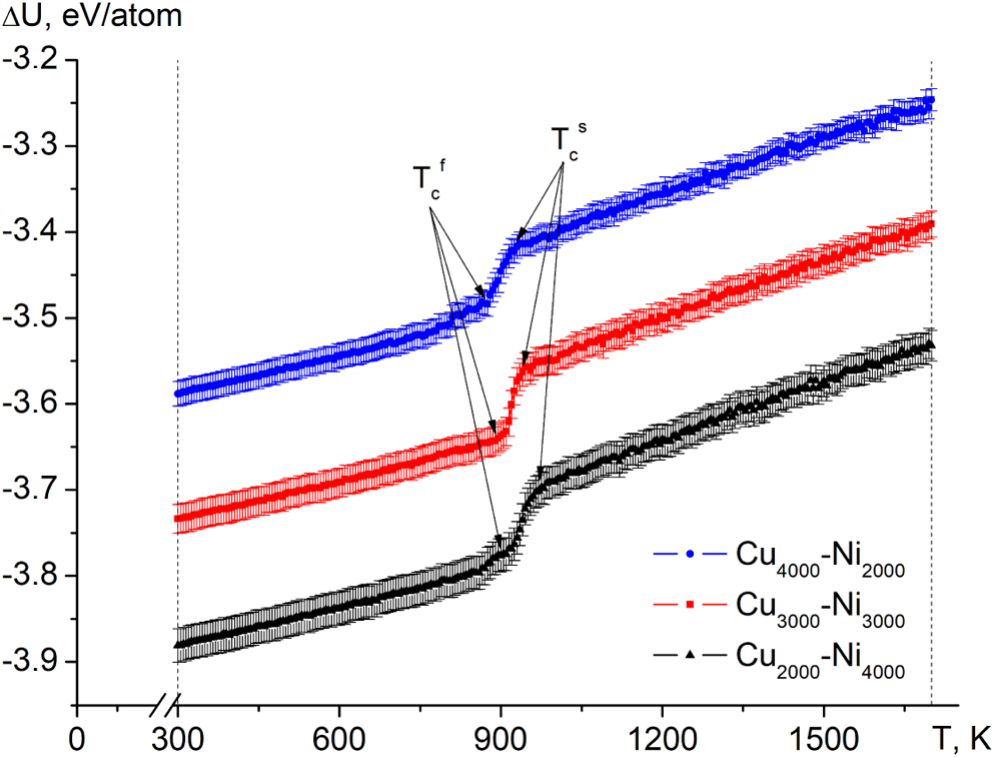
Results of MD simulations of the behavior of the Cu–Ni system during the cooling, showing caloric dependences of the potential part of the internal energy for binary nanoparticles Cu–Ni of various compositions.

Figure 4 illustrates the caloric dependencies of the potential component of the internal energy for binary nanoparticles with various compositions. The hysteresis type of such dependences is characteristic of metallic nanoparticle melting processes and is observed both in computer experiments^72^ and in synthesis.^73^

The simulation results revealed a phase transition corresponding to the crystallization of the nanoparticles. Notably, for each system studied, we observed distinct temperatures marking the onset of the crystallization process *T*_c_^s^ and its completion *T*_c_^f^, which coincides with our previous works.^72,74,75^ Importantly, we observed that these values (*T*_c_^s^ and *T*_c_^f^) are composition dependent (Figure 4 and Table 3).

**Table 3:** Values of Characteristic Temperatures Describing the Hysteresis of the Crystallization Process of Cu–Ni NPs

composition	*T*_c_^s^, K	*T*_c_^f^, K	Δ*T*, K
Cu_4000_–Ni_2000_ (2:1)	910	860	50
Cu_3000_–Ni_3000_ (1:1)	945	905	40
Cu_2000_–Ni_4000_ (1:2)	990	910	80

From the presented data, one can see, in contrast to the systematic increase in the values of *T*_c_^s^ and *T*_c_^f^ with an increase in the proportion of nickel atoms in the nanoparticles, the nature of the change in the value of Δ*T* = *T*_c_^s^ – *T*_c_^f^ (representing the temperature range of the crystallization process) does not follow the same trend. Notably, the lowest value of Δ*T* is reached when the NPs have an equiatomic composition.

Thus, the equiatomic Cu–Ni mixture (Cu_3000_–Ni_3000_) shows the lowest Δ*T* (40 K), since such mixtures often exhibit a more uniform atomic distribution and mixing behavior. In equiatomic alloys, the interactions between Cu and Ni atoms are more balanced, which can lead to more uniform nucleation and crystallization processes. As a result, the temperature difference between the onset of crystallization (*T*_c_^s^) and the end of crystallization (*T*_c_^f^) is smaller, which reduces the hysteresis (Δ*T*). This suggests a more stable crystallization path compared to nonequiatomic compositions, where the atomic interactions are no longer balanced, leading to greater hysteresis and a wider crystallization range.

**Figure 5:**
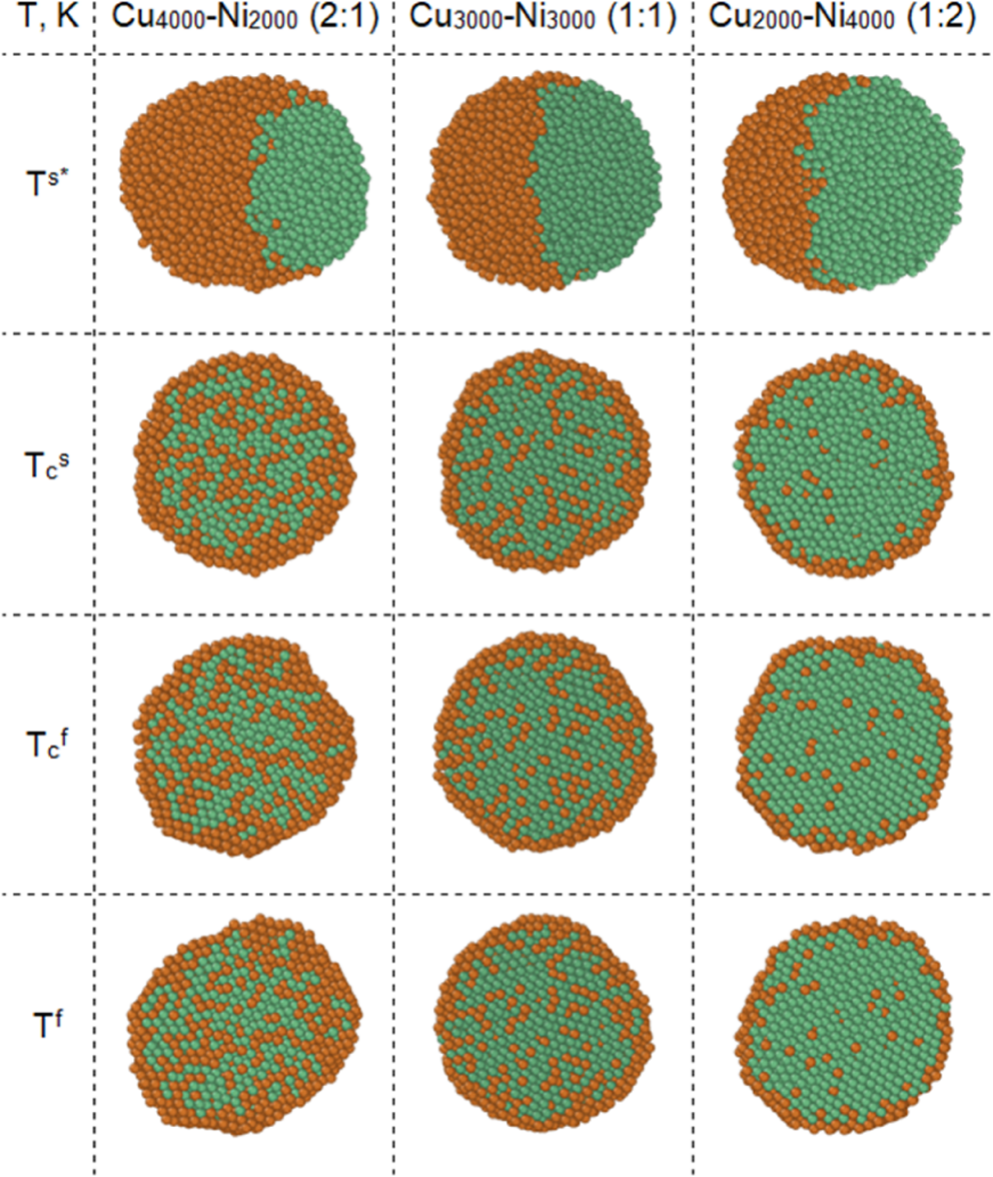
Results of molecular dynamics configurational simulations. Instantaneous configurations of Cu–Ni NPs at different temperatures. *Configurations are shown after relaxation. Brown, Cu atoms; green, Ni atoms.

Figure 5 shows instantaneous configurations (corresponding to equatorial sections) of the NP composition Cu_4000_–Ni_2000_ (2:1), Cu_3000_–Ni_3000_ (1:1), and Cu_2000_–Ni_4000_ (1:2), corresponding to the temperatures of the beginning of the crystallization process *T*_c_^s^ and its completion *T*_c_^f^. As a result of the process of coalescence and further diffusion of atoms for NPs enriched with copper atoms (Cu_4000_–Ni_2000_ (2:1)), as expected, the fraction of internal atoms is large, although the shell is formed from 2 to 3 monolayers. In addition, at a temperature of 300 K, the NP has a significantly aspherical shape, which indicates the ongoing process of segregation and the nonequilibrium state of the NP. For the Cu_3000_–Ni_3000_ NPs (1:1), surface segregation of copper atoms is observed even before the start of the crystallization process. With a further decrease in temperature, the core–shell structure becomes more defined and the number of copper atoms observed in the central part of the NP decreases. In the case of an NP enriched with nickel atoms (Cu_2000_–Ni_4000_ (1:2)), a shell of copper atoms is formed already at a temperature corresponding to the onset of crystallization, and subsequently, the structure of the NP and the proportion of internal copper atoms remain practically unchanged. Consistent with previous computational investigations,^76,77^ our findings reveal similar structural behavior in Cu–Ni nanoparticles. Notably, thermodynamic and MD simulations converge in their predictions, suggesting the preferential segregation of Cu atoms toward the surface of Cu–Ni nanoparticles. This segregation leads to the formation of a low-level core–shell structure, characterized by incomplete segregation of components and a less distinct boundary between the core and shell regions. Investigation into the sintering behavior of dissimilar element nanoparticles^66^ reveals that elements with lower melting temperatures exhibit a predominant role in diffusion processes. Consequently, the final morphology of the sintered nanoparticles is primarily determined by these elements due to their enhanced diffusion rates. However, the temperature range investigated was limited to 800 K, which precluded the observation of the core–shell structure formation. An alternative approach was applied to investigate the surface segregation of copper atoms. The initial configuration of the binary system consisted of either a uniform distribution of components or a core–shell structure, with copper atoms forming the core. Notably, for a nearly equiatomic binary particle (Cu_49_Ni_51_, 2899 atoms, ∼4 nm), approximately 78% of copper atoms segregate to the surface at the melting temperature.^78^

Based on Figure 1, it is impossible to definitively determine the structure of individual agglomerates smaller than 5 nm. Thus, MD modeling in this case serves a predictive function, and at these scales, it predicts the surface segregation of Cu. At scales around 5 μm, considering the porosity of the obtained samples and based on the EDX element mapping, it is reasonable to conclude a highly homogeneous distribution of both Cu and Ni atoms.

**Figure 6:**
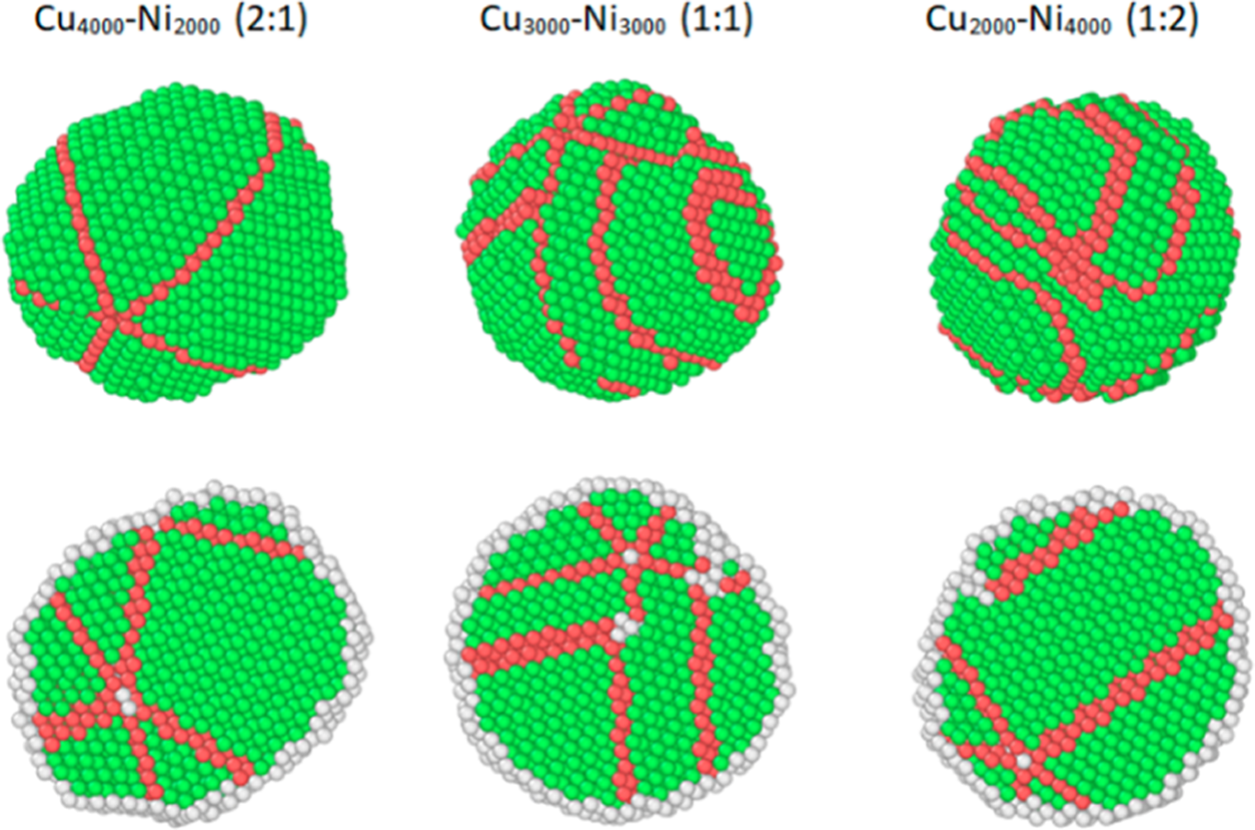
Final configurations of the NPs. The top row shows bulk nanoparticles with only recognized local structures; the second row shows equatorial sections of these nanoparticles including disordered atoms. Green—fcc, red—hcp, and white—unrecognized.

The final configurations for the NPs of the different compositions at *T*_f_ were analyzed and visualized using Ovito software^79^ using the polyhedral template matching tool^80^ and presented in Figure 6. From the obtained data one can see that for NP with an increased number of copper atoms, the final crystal structure corresponds to fifth-order symmetry and it is generally characteristic of both copper single crystals and mono- and binary nanoparticles of other fcc metals.^81,82^ To the best of our knowledge, there are no experimental works or computer simulation results for binary Cu–Ni NPs in which pentagonal symmetry was observed. At the same time, for Ni NPs, the pentagonal symmetry was experimentally observed, but with a high probability within the framework of synthesis by liquid phase plasma processing.^83^ In our case, however, the possibility of the formation of fifth-order symmetry for binary Cu–Ni NPs has yet to be confirmed experimentally. We did not observe crystals with such symmetry in TEM images, which may be related to the fact that the conditions in real synthesis, like temperature, kinetics of the reaction, etc., are insufficient to obtain or preserve NPs with fifth-order symmetry. For the NPs with an equiatomic composition and a composition enriched with nickel atoms, a face-centered cubic (fcc) structure with twinning planes, identified as hexagonal close-packed lattices (hcp), is formed.

Overall, based on the presented MD results, we can conclude that the segregation scenario, and hence the thermal stability, depends on the composition of the NPs.^84^

## 4 Discussion

The SCS is a complex synthesis process based on a highly exothermic self-propagating redox reaction of initial components, mixed in solution. Typically, SCS is thermally activated and the synthesis process of metallic NPs can be divided into several main stages: drying (initial heating with removal of liquid solvent in case of water), combustion (decomposition of the initial components, initiation and propagation of the combustion reaction), reduction (transformation of the initial amorphous metal oxides into nitrides and their subsequent decomposition into metal NPs), and cooling down (postcombustion stabilization of the final product, thermal-activated crystal growth, reoxidation, etc.).^51,52,60^ Due to the nature of the exothermic self-propagating reaction, the combustion, reduction, and cooling down stages of the SCS progress in parallel in different local volumes of the reacting system.

It is known^17,29,85^ that the Cu atoms tend to segregate from the alloy crystal cell and form separate clusters. In fact, in our previous paper,^60^ we discussed the possibility of the formation of small crystallites of interconnected but separate Cu–Ni phases. A distinctive feature of ref (60) is the use of an alternative modeling method—the Monte Carlo method. Furthermore, the data analysis of nanoparticle characteristics showed a close integration of Cu and Ni crystalline structures, which tend to form a bimetallic alloy. Regarding the investigation,^86^ even smaller nanoparticles were studied, with rather high MD simulation rates of 1 K/ps. Additionally, the local structure was not analyzed in that study and the correct parameters for the EAM potential were not provided, making it difficult to accurately reproduce the experiment. It is also worth noting that, based on Figure 4 of that work, no pronounced surface segregation of copper was observed in the Cu682–Ni682 system. This could be due to the high cooling rate used.

In the current study, we also found separate Cu and Ni grains but only in one instance for the CuNi sample (Figure S2), as the majority of crystals are clearly bimetallic. The mechanisms behind the formation of the bimetallic or separated metallic crystallites are not clear. We consider three possible phase formation scenarios: (i) clustering of the atoms of the same type in an initial solution or the stage of gel-formation; (ii) clustering and atoms reorganization after the completion of the high-temperature stage of combustion reaction during the cooling stage; and (iii) mixed mechanism—where the initial formation of the cluster occurs in solutions/gels and develops during the combustion/cooling. In the case of the first and third scenarios, the clusters form before the combustion reaction and then can evolve during most of the synthesis stages, with the highest intensity at high temperature. In the case of the second scenario, however, the clustering should mostly occur during the short high-temperature stage of combustion, when the system has the highest energy state and the atoms are the most mobile. In any case, it is important that in all three scenarios, if clusters form, they should be present at the high-temperature stage of a combustion reaction.

The results of the MD simulations showed that in the case of the presence of an initial metallic phase segregation at high temperatures, the clustering keeps progressing further with the formation of quasi-core–shell structures. The tendency of Cu atoms to migrate on the surface of the nanoparticles was described before.^26^ More important, however, is that in the case of a preexisting segregated structure the energetic state of the system does not allow the formation of bimetallic crystals, as it would require additional energy input for system homogenization and time for its stabilization. In contrast, simple evolution of separate Cu- and Ni-based crystal phases is more energetically favorable. This means that the bimetallic crystallites, observed by TEM, were not segregated at the high-temperature stage of combustion but rather performed as they are during the early stages, like Me-fuel complexes formation and then crystallized during the combustion.^52,87^ At the same time, the fast heating–cooling rate of SCS prevented phase segregation and significant atom clustering. This also means that the second scenario of the cluster formation is unlikely, as in this case there would be observed a lot of core–shell structures rather than bimetallic crystallites (Figure 3) or separate Cu–Ni crystals (Figures S2–S4).

The first scenario is more realistic, as the presence of preformed segregated structures before the high-temperature stage of the reaction is necessary. However, the results of MD simulations showed that even at very fast cooling the nanoparticles keep evolving due to the tendency for clustering as a system energy minimalization mechanism – the third scenario. Thus, we suggest that both factors: the preformation of a mixed or clustered system in the early stages of the synthesis, and the reaction kinetic, i.e., the time of existence of the system at high temperature with the energy of the system high enough for noticeable atoms mobility, play a significant role.

## 5 Conclusions

In summary, we successfully used the SCS method for obtaining bimetallic Cu–Ni nanoparticles of Cu2Ni, CuNi, and CuNi2 chemical composition. The method used allows the synthesis of fine nanoparticles of 12–18 nm with a high degree of phase homogeneity with no significant atom clustering indicated, alongside phase-segregated nanoparticles predicted by molecular dynamics simulations. The formation of the homogeneous bimetallic structures was attributed to the solution-based precursor’s preparation and fast phase formation during the high-temperature self-combustion synthesis process and product cooling.

There were conducted molecular dynamic computer simulations for similar concentration compositions but in the size range on the order of 4.5–5.5 nm. The simulation results predicted surface segregation of copper atoms during cooling of the system from 1700 to 300 K in the case of initial Cu–Ni phase segregation at high temperature. It was shown that the segregation scenario and hence the thermal stability depend on the composition of the NPs. The calculations revealed the possibility of forming a structure with pentagonal symmetry in binary Cu–Ni NPs enriched with copper atoms.

The comparison of the experimental and computer simulation results allows us to formulate a possible scenario for the formation of the initial Cu–Ni phase segregation, which includes clustering of the atoms of the same type in an initial solution or the stage of gel-formation and further developing segregation during the combustion/cooling stage. Based on that we suggested that the preformation of a mixed or clustered system in the early stages of the synthesis, and the reaction kinetics, i.e., the time of existence of the system at high temperature with the energy of the system high enough for noticeable atom mobility, play a significant role in the degree of phase homogeneity of MPEMs.

The results of the study are important for the development of the method of synthesis of alloys and others with MPEMs and understanding of the behavior and properties of such materials.

## Data Availability

Data will be made available on request.

## Supporting Information Available

Initial chemical compositions of all samples; example of the initial configuration of atoms; HR TEM, HAADF STEM, and SAED images of the CuNi sample. (PDF)
