# Supplementary material for: Bimetallic CuNi Nanoparticle Formation: Solution Combustion Synthesis and Molecular Dynamic Approaches
Source: Inorg Chem. 2024 Dec 16;63(52):24844–54. doi: 10.1021/acs.inorgchem.4c04260 (PMC11688667; doi:10.1021/acs.inorgchem.4c04260)
Supplement: Supplementary file 1 — ic4c04260_si_001.pdf [file ic4c04260_si_001.pdf]

Supporting Information

Bimetallic CuNi nanoparticle formation: solution combustion synthesis and molecular dynamic approaches

Valentin Romanovski [1,2,*], Nickolay Sdobnyakov [3], Sergey Roslyakov[2], Andrei Kolosov [3], Kirill Podbolotov [4,2], Kseniya Savina[3], Witold Kwapinski [5], Dmitry Moskovskikh[2], Alexander Khort[6,*]

[1] *Department of Materials Science and Engineering, University of Virginia, Charlottesville, USA.*

[2] *Science and Research Centre of Functional Nano-Ceramics, National University of Science and Technology "MISIS", Moscow, Russia*

[3] *Department of General Physics, Tver State University, Tver, Russia*

[4] *Physical-Technical Institute of the National Academy of Sciences of Belarus, Minsk, Belarus*

[5] *Department of Chemical Sciences, Bernal Institute, University of Limerick, Limerick, Ireland*

[6] *Division Surface and Corrosion Science, KTH Royal Institute of Technology, Stockholm, Sweden*

*correspondence to: rvd9ar@virginia.edu (V. Romanovski), khort@kth.se (A.Khort)

Table S1. Initial chemical compositions of the experimental samples, calculated for the synthesis of the 1 g of the final metallic product.

Samples	Initial compositions, g		
	$Cu(NO_3)_2 \cdot 3H_2O$	$Ni(NO_3)_2 \cdot 6H_2O$	$C_2H_5NO_2$
Cu2Ni	2.60	1.56	2.83
CuNi	1.98	2.38	2.86
CuNi2	1.33	3.21	2.90

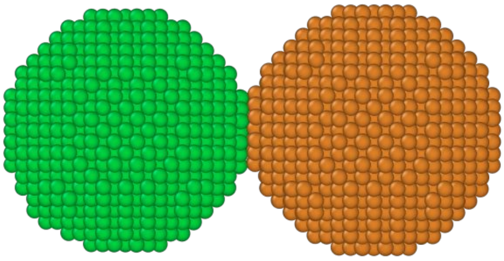

Fig. S1. An example of the initial configuration of Cu_{3000}-Ni_{3000} (1:1) NPs (hereinafter, copper atoms are shown in brown, nickel atoms in green)

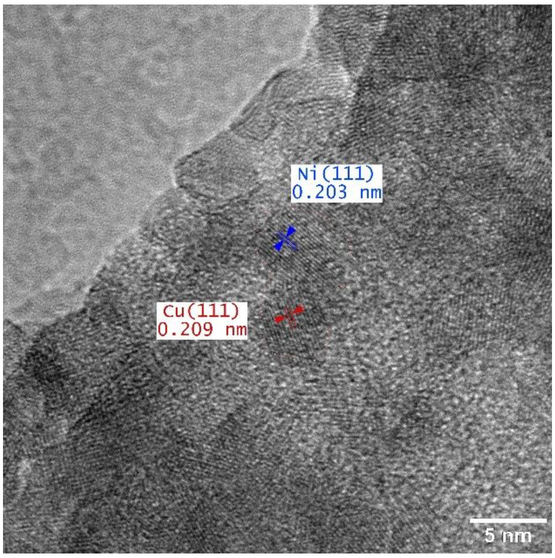

Fig S2. TEM image of the CuNi NPs. The image shows a split Cu and Ni crystallites

The HAADF STEM image of a particle and corresponding elemental distribution maps (EDX mapping) are presented in Fig. S3. Two areas with different distribution of elements can be distinguished, which is confirmed by quantitative EDX analysis of marked by squares areas in Fig. S3a (Table S2). The areas marked by squares 1 and 3 have increased concentrations of Ni and the area 2 has near equal distribution. Moreover, detailed analysis of areas 1, 3 demonstrates clustering of Cu (marked by arrow in Fig. S3d).

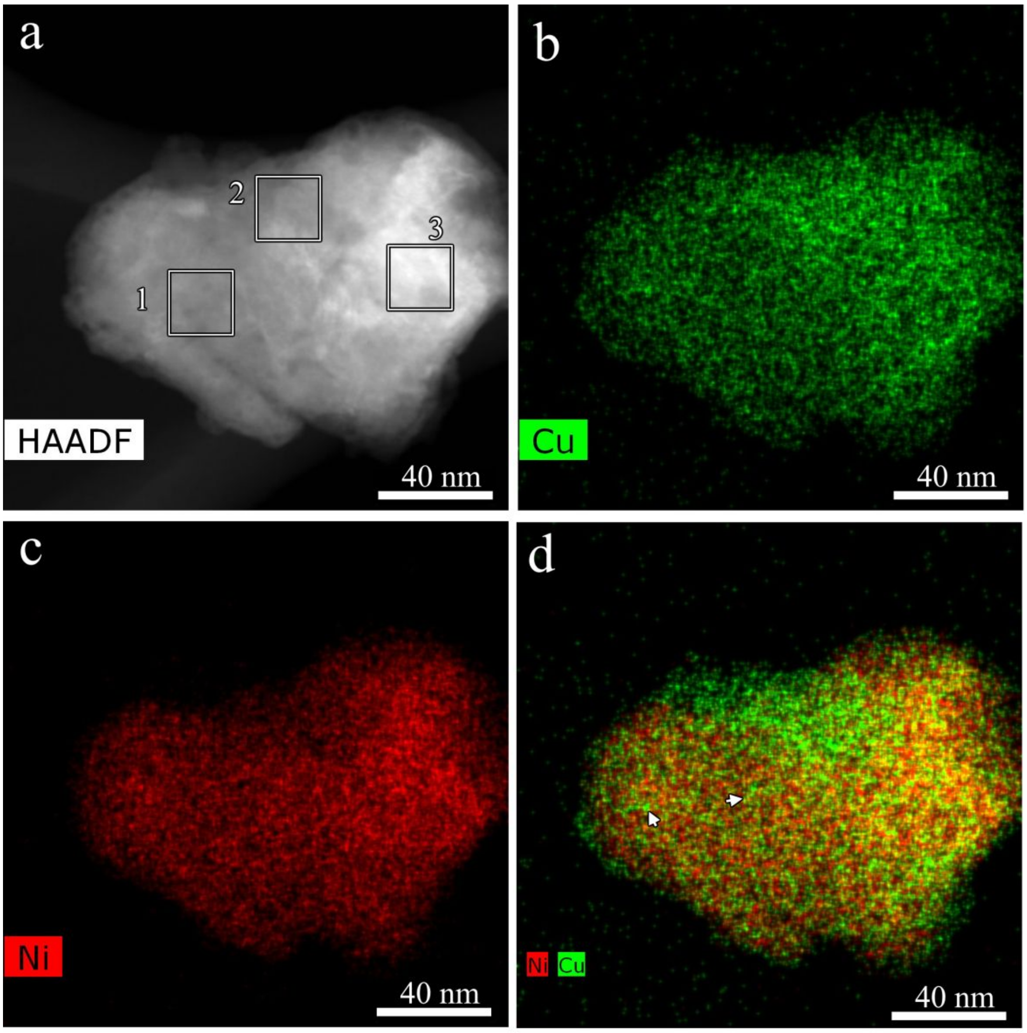

Fig S3. (a) HAADF STEM image, and (b-d) EDX mapping of NPs of the CuNi sample.

Table S2. Quantitative EDX analysis of areas 1, 2 and 3 in Fig. S3a

Element	Area 1, at. %	Area 2, at. %	Area 3, at. %
Cu	30.99	53.27	31.45
Ni	69.01	46.73	68.55

Selected area electron diffraction (SAED) pattern indexed in terms of the FCC structure evidence this is a single particle and not an agglomerate of particles (Fig. S4). However, parts of rings are visible instead of sharp spots, which is an attribute of many low-angle rotated parts of the crystal. In addition, one can see a doubling of those elongated spots (marked by an arrow in Fig. S4) because of the presence of two structures with close parameters. We suppose uneven distribution of elements in particles leads to the formation of areas with slightly different unit cell parameters. Low-angle rotation occurs because of mechanisms of elastic stress relaxation.

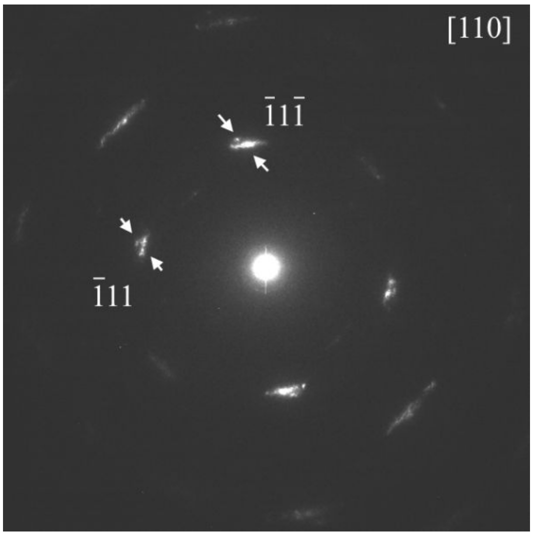

Fig S4. SAED pattern of the NP of CuNi sample, demonstrated in Fig. S3.
